# The effects of reward and punishment on the performance of ping-pong ball bouncing

**DOI:** 10.3389/fnbeh.2024.1433649

**Published:** 2024-06-27

**Authors:** Cong Yin, Yaoxu Wang, Biao Li, Tian Gao

**Affiliations:** ^1^School of Kinesiology and Health, Capital University of Physical Education and Sports, Beijing, China; ^2^School of Recreation and Community Sport, Capital University of Physical Education and Sports, Beijing, China

**Keywords:** reward, punishment, real-world motor skill learning, ping-pong ball bouncing, motor learning, motor memory, motor transfer

## Abstract

**Introduction:**

Reward and punishment modulate behavior. In real-world motor skill learning, reward and punishment have been found to have dissociable effects on optimizing motor skill learning, but the scientific basis for these effects is largely unknown.

**Methods:**

In the present study, we investigated the effects of reward and punishment on the performance of real-world motor skill learning. Specifically, three groups of participants were trained and tested on a ping-pong ball bouncing task for three consecutive days. The training and testing sessions were identical across the three days: participants were trained with their right (dominant) hand each day under conditions of either reward, punishment, or a neutral control condition (neither). Before and after the training session, all participants were tested with their right and left hands without any feedback.

**Results:**

We found that punishment promoted early learning, while reward promoted late learning. Reward facilitated short-term memory, while punishment impaired long-term memory. Both reward and punishment interfered with long-term memory gains. Interestingly, the effects of reward and punishment transferred to the left hand.

**Discussion:**

The results show that reward and punishment have different effects on real-world motor skill learning. The effects change with training and transfer readily to novel contexts. The results suggest that reward and punishment may act on different learning processes and engage different neural mechanisms during real-world motor skill learning. In addition, high-level metacognitive processes may be enabled by the additional reinforcement feedback during real-world motor skill learning. Our findings provide new insights into the mechanisms underlying motor learning, and may have important implications for practical applications such as sports training and motor rehabilitation.

## 1 Introduction

Seeking rewards and avoiding punishment are powerful motivators that influence human behavior. Reward- and punishment-based feedback has been studied extensively in various fields, including psychology (Thorndike, 1933), artificial intelligence (Kaelbling et al., 1996), robotics (Kormushev et al., 2003), and neuroeconomics (Glimcher et al., 2009). However, until only recently, the ways in which reward and punishment specifically influence human motor learning have not been extensively studied.

Motor skill learning, one of the main categories of motor learning, generally refers to the neural changes that allow an organism to perform a motor task better, faster, or more accurately than before (Diedrichsen and Kornysheva, 2015). In the real world, many motor skills are extremely complex and require practice over thousands of hours (Krakauer et al., 2019). However, the laboratory-based motor skill tasks used to study the reinforcement effects are typically simple, and participants practice the tasks only for only one session within a single day. It is unknown whether the effects of reward and punishment on real-world motor skill learning change with training.

Punishment has been found to benefit online performance for different types of motor tasks, ranging from motor adaptation (Galea et al., 2015; Song and Smiley-Oyen, 2017; Hill et al., 2020; Yin et al., 2023a), motor skill learning (Wächter et al., 2009; Steel et al., 2016, 2020), and reinforcement-based motor learning (Song et al., 2020; Yin et al., 2023b), possibly through the loss aversion mechanism indicated in prospect theory (Kahneman and Tversky, 1979). In particular, for motor adaptation, punishment improves online performance, but impairs memory retention (Hill et al., 2020). For the serial reaction time task (SRTT), punishment benefits sequence knowledge during early learning (Steel et al., 2016, 2020). For a reinforcement-based visuomotor task, punishment promotes initial learning, but impairs later learning in the new direction (Yin et al., 2023b). It is possible that punishment only benefits the initial stage of motor learning. Therefore, we predict that punishment will promote early learning, but not late learning, in complex real-world motor skill tasks.

In contrast, reward has been found to promote motor memory retention, but not benefit online performance (Abe et al., 2011; Galea et al., 2015). In visuomotor adaptation, reward does not accelerate initial learning, but promotes relearning of the same task (Song and Smiley-Oyen, 2017). In reinforcement-based visuomotor learning, reward has been found to promote relearning of the same task in a new direction (Yin et al., 2023b). It appears that reward begins to work as participants become skilled at a task. Therefore, we predict that although reward may not benefit early learning, it will promote late learning for complex real-world motor skill learning.

In addition to learning, motor transfer, the analysis of how learning in one context influences performance in untrained contexts provides a unique window for investigating the nature of motor learning (Poggio and Bizzi, 2004; Shadmehr, 2004). The effects of reward and punishment may not be limited to the context in which people receive the feedback, but transfer to untrained contexts. However, previous studies have focused on the process of online learning and offline memory. Few studies have examined the transfer effect of reward and punishment in motor learning (but see Yin et al., 2023a,b). In motor adaptation, the effect of combining reward and punishment is surprisingly found to transfer to opposite rotation learning, during which meta-learning process is supposed to be activated (Yin et al., 2023a). Similarly, we hypothesize that the effect of reward and punishment can be readily transferred to untrained contexts.

The present study aims to investigate the effects of reward and punishment on complex real-world motor skill learning, which requires longer training time than simple laboratory-based motor skill tasks. Based on the aforementioned inferences, we hypothesize that reward and punishment have differential effects on real-world motor skill learning: they may change with training and transfer to novel contexts. Specifically, we predict that punishment will promote early learning, whereas reward will promote late learning. To test the hypothesis, we trained and tested three groups of novice participants on a ping-pong ball bouncing task for three consecutive days. Table tennis is not only a popular Olympics sport, but also a good choice for everyday exercise. Bouncing a ball on a paddle is a basic training for table tennis and helps to develop a “feel” for the ball. The training and test sessions were identical across the 3 days: participants were trained with their right (dominant) hand each day under conditions of either monetary reward, monetary punishment, or a neutral control condition (neither). Before and after the training session, participants were tested with their right hand and then with their left hand without any motivational feedback.

## 2 Materials and methods

### 2.1 Participants

The experiment included 48 right-handed participants randomly assigned to the reward group (23.4 ± 1.5 years), the punishment group (23.6 ± 1.0 years) and the control group (23.0 ± 1.3 years), with 16 individuals (half men and half women) in each group. All participants had no ping-pong training background, signed an institutionally approved informed consent form, were naive to the purpose of the study, and were compensated for their participation. The Institutional Review Board of the Capital University of Physical Education and Sports approved all experimental procedures.

### 2.2 Basic movements

**Figure 1:**
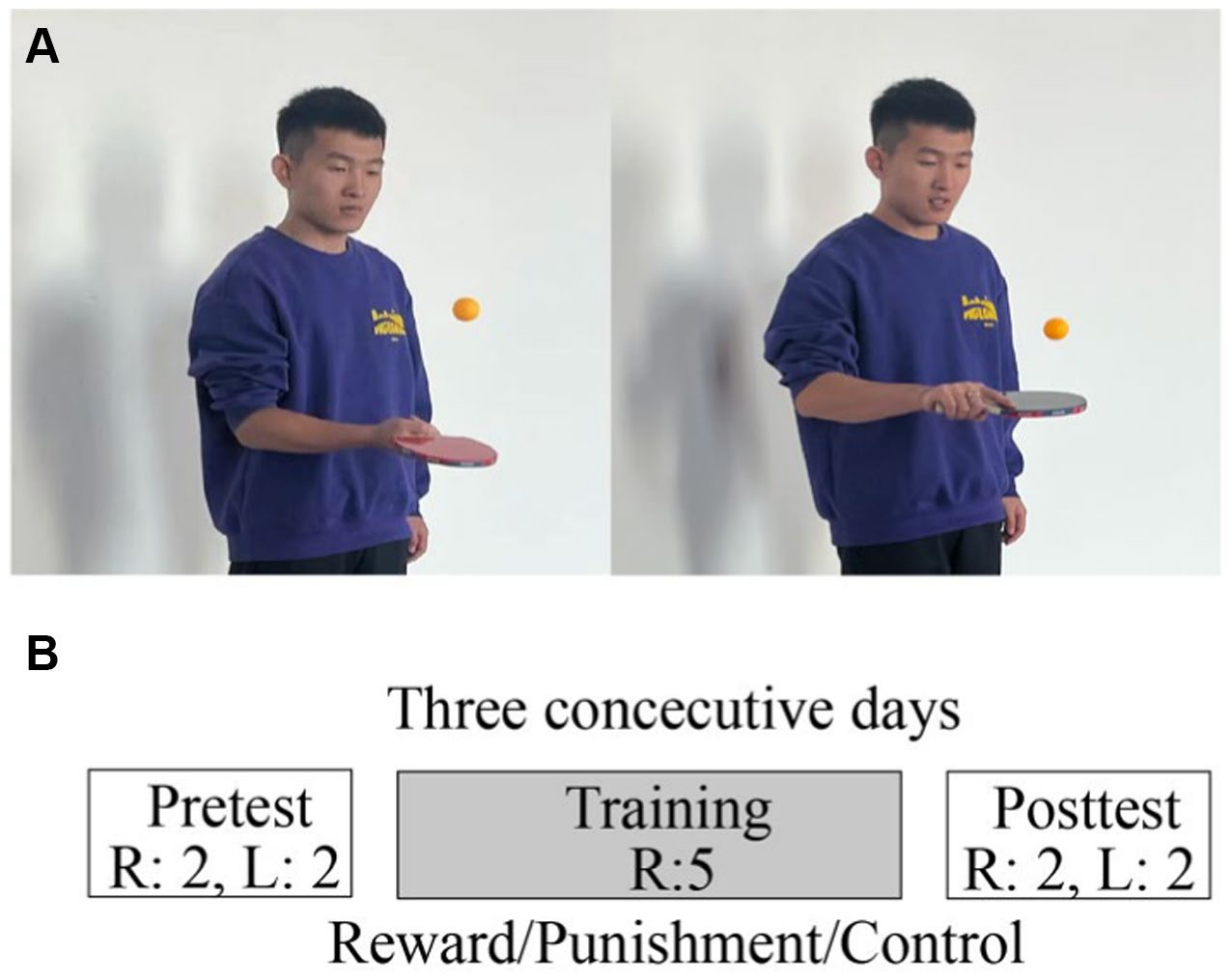
The basic movement of the ping-pong ball bouncing task **(A)** and the experimental design **(B)**. R stands for right hand and L for left hand. The number represents the number of trials in each phase.

Participants were instructed to bounce a ping-pong ball in the air with a paddle within 30 s. They were first instructed on the correct posture for holding the paddle. During the 30 s, they should stand still and not move their feet. They were asked to take turns hitting the ball with both sides of the paddle, i.e., to rotate their wrists after each hit (Figure 1A). As soon as the ball fell on the group or the participants moved their feet, they stopped hitting the ball and the current trial ended. One experimenter counted the time with a timer and the other experimenter counted and recorded the number of times they hit the ball in the current trial. Participants were instructed to hit the ball as many times as possible in the limited time available. After each trial, participants were told the number of times they hit the ball and were given a short break (less than 1 min).

### 2.3 Experimental procedures

The experiment lasted for three consecutive days, and the procedures were identical for all 3 days (Figure 1B). On each day, the experiment was divided into three consecutive phases: pretest, training, and posttest. The procedures during pretest and posttest were identical: the participants bounced the ball first with their right hand and then with their left hand for 2 trials each. During the training phase, participants bounced the ball with their right hand for 5 trials. Only during the training phase did the reward and the punishment groups receive different reinforcement feedback depending on the experimental condition (except for this phase, the task was identical for all three groups). The two groups were informed about the scoring rule before the experiment:

Reward. 5 points: bouncing number ≤ 10; 10 points: bouncing number > 10 and ≤25; 15 points: bouncing number > 25 and ≤40; 20 points: bouncing number > 40.

Punishment. −20 points: bouncing number ≤ 10; −10 points: bouncing number > 10 and ≤25; −5 points: bouncing number > 25 and ≤40; 0 points: bouncing number > 40.

Both groups began the training phase with 0 points, and the points were accumulated over the 3 days of the phase. After each training trial, a point card was placed in front of the participant. After the training phase, the reward and the punishment participants received 5 different cards representing the 5 points for each trial. The reward group earned money based on the points accumulated over the 3 days (winning 1 yuan for every 10 positive points), while the punishment group lost money based on the negative points accumulated (losing 1 yuan for every 10 negative points). The reward group started with 15 yuan and won about 10–20 yuan. The punishment group started with 45 yuan and lost about 10–20 yuan. Both groups received on averaged about 30 yuan. Each group was explicitly instructed about both the point-number relationship and the maximum points and money they could win or lose during the training phase.

### 2.4 Data analysis

The study analyzed the number of ball bounces in each trial. During the pretest and posttest phases, we averaged the data for two right-handed trials and two left-handed trials. During the training phase, because we did not find a steady trend of increase, we averaged the data of the five training trials. A 3 (groups) × 3 (days) mixed design ANOVA was performed on the training data to compare the effects of reward and punishment on motor learning. A 3 (groups) × 3 (days) × 2 (tests) mixed design ANOVA was performed on the right-hand test data to compare the effects of reward and punishment on motor memory, both for short-term memory across the phases and long-term memory across the days. Similarly, a 3 (groups) × 3 (days) × 2 (tests) mixed design ANOVA was performed on the left- hand test data to test whether the effects of reward and punishment could transfer to the untrained scenario.

All *post hoc* comparisons of means were performed using Bonferroni’s correction for multiple comparisons. Normality assumptions were tested prior to conducting *t*-tests and ANOVA, and all dependent variables met these assumptions. Mauchly’s test of sphericity was used to test for homogeneity of variance in mixed-model ANOVAs. Greenhouse–Geisser corrections were applied when sphericity tests revealed unequal variance, where significant effects were robust to heteroscedasticity. The significance level was set at *α* = 0.05. Data are presented as mean ± standard error (SE) across participants. All analyses were performed in SPSS (version 26.0, IBM Corp., Armonk, NY, United States).

## 3 Results

Before examining the effects of reinforcement on motor learning, we first confirmed that the three groups started from a similar level before training. During the pretest phase on day 1, the number of ball bounces for the right hand was 11.3 ± 1.3, 11.8 ± 1.1, and 11.9 ± 1.5 for the reward, punishment and control groups, respectively. One-way ANOVA showed no significant difference among the groups [*F*_(2, 45)_ = 0.13; *p* = 0.88, $\eta_{p}^{2}$ = 0.01]. For the left hand, the number of ball bounces was 5.7 ± 0.4, 5.4 ± 0.4, and 6.4 ± 0.6 for the reward, punishment and control groups, respectively. Similarly, no significant difference was found [*F*_(2, 45)_ = 1.52; *p* = 0.23, $\eta_{p}^{2}$ = 0.06]. All groups showed better performance in bouncing the ping-pong ball with the right hand than with the left hand [paired samples *t*-test: *t*_(15)_ = 4.39, 5.54, and 3.93; *p* = 0.001, <0.001, =0.001; *d* = 1.57, 2.07, and 0.99]. These results suggest that the three groups showed similar initial levels of ping-pong ball bouncing, regardless of whether the superior right or left hand was used.

### 3.1 The effects of reward and punishment on motor learning

**Figure 2:**
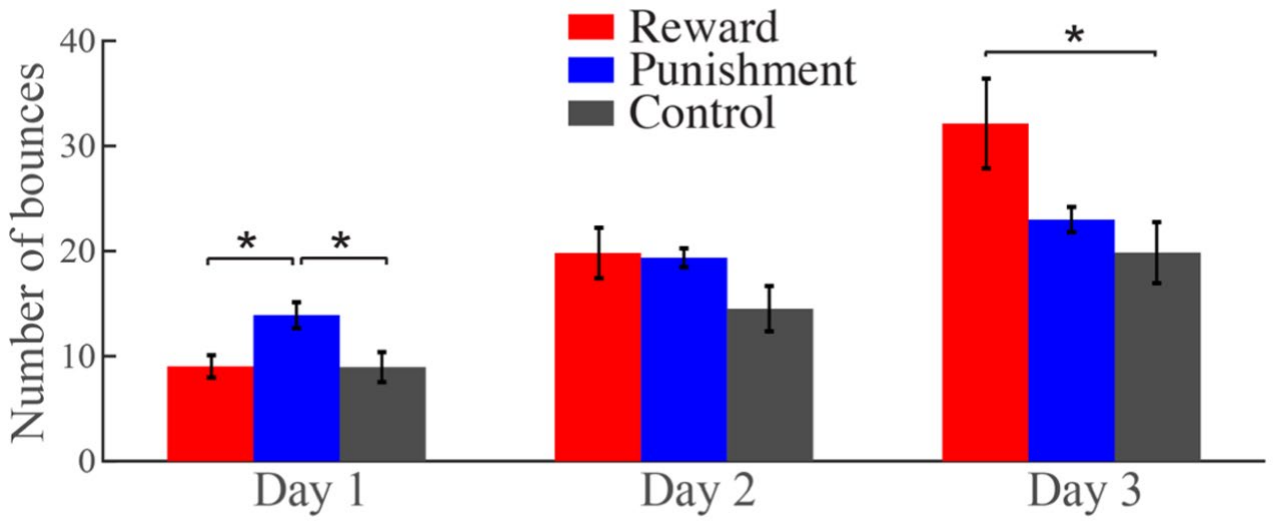
The effects of reward and punishment on online motor learning. Error bars denote SEM. The same is below. *p < 0.05.

On day 1, the average number of ball bounces during the training phase was 9.0 ± 1.1, 13.9 ± 1.2, and 9.0 ± 1.4 for the reward, punishment and control groups, respectively. On day 2, the average number of ball bounces was 19.8 ± 2.4, 19.4 ± 0.9, and 14.5 ± 2.2 for the three groups, respectively. On day 3, the average number of ball bounces was 32.1 ± 4.3, 23.0 ± 1.2, and 19.8 ± 2.9 (Figure 2). Accordingly, the average point for the reward group was 6.8 ± 0.4, 10.7 ± 0.8, and 13.9 ± 1.0, whereas that for the punishment group was −14.1 ± 0.9, −9.9 ± 0.4, and −8.6 ± 0.6 over the 3 days. Reward increased while punishment decreased as performance improved from day 1 to day 3.

A 3 (groups) × 3 (days) mixed-design ANOVA on the training data revealed a significant main effect of day [Greenhouse–Geisser adjustment: *F*_(1.41, 63.45)_ = 74.75; *p* < 0.001, $\eta_{p}^{2}$ = 0.62] and a significant interaction between group and day [Greenhouse–Geisser adjustment: *F*_(2.82, 63.45)_ = 7.13; *p* < 0.001, $\eta_{p}^{2}$ = 0.24]. The main effect of group was not significant [*F*_(2, 45)_ = 2.66; *p* = 0.081, $\eta_{p}^{2}$ = 0.11]. Specifically, the number of ball bounces increased from day to day for the reward group (comparison between day 2 and day 1: mean difference = 10.8, *p* < 0.001; comparison between day 3 and day 2: mean difference = 12.3, *p* < 0.001). The control group showed a similar learning tendency as the reward group (comparison between day 2 and day 1: mean difference = 5.5, *p* = 0.001; comparison between day 3 and day 2: mean difference = 5.3, *p* = 0.03). However, the punishment group showed a different learning tendency from the two groups: learning increased significantly from day 1 to day 2 (mean difference = 5.5, *p* = 0.001), whereas performance did not increase from day 2 to day 3 (mean difference = 3.6, *p* = 0.20).

We then directly compared the learning of the three groups on each day. On day 1, the learning effect of the punishment group was superior to that of the reward group (mean difference = 4.9, *p* = 0.03) and the control group (mean difference = 4.9, *p* = 0.02). On day 2, there was no difference among the three groups. However, on day 3, the learning of the reward group was significantly better than that of the control group (mean difference = 12.3, *p* = 0.02). The results suggest that reward and punishment have differential effects on motor learning: punishment promotes early learning, while reward promotes late learning.

### 3.2 The effects of reward and punishment on short-term and long-term motor memory

**Figure 3:**
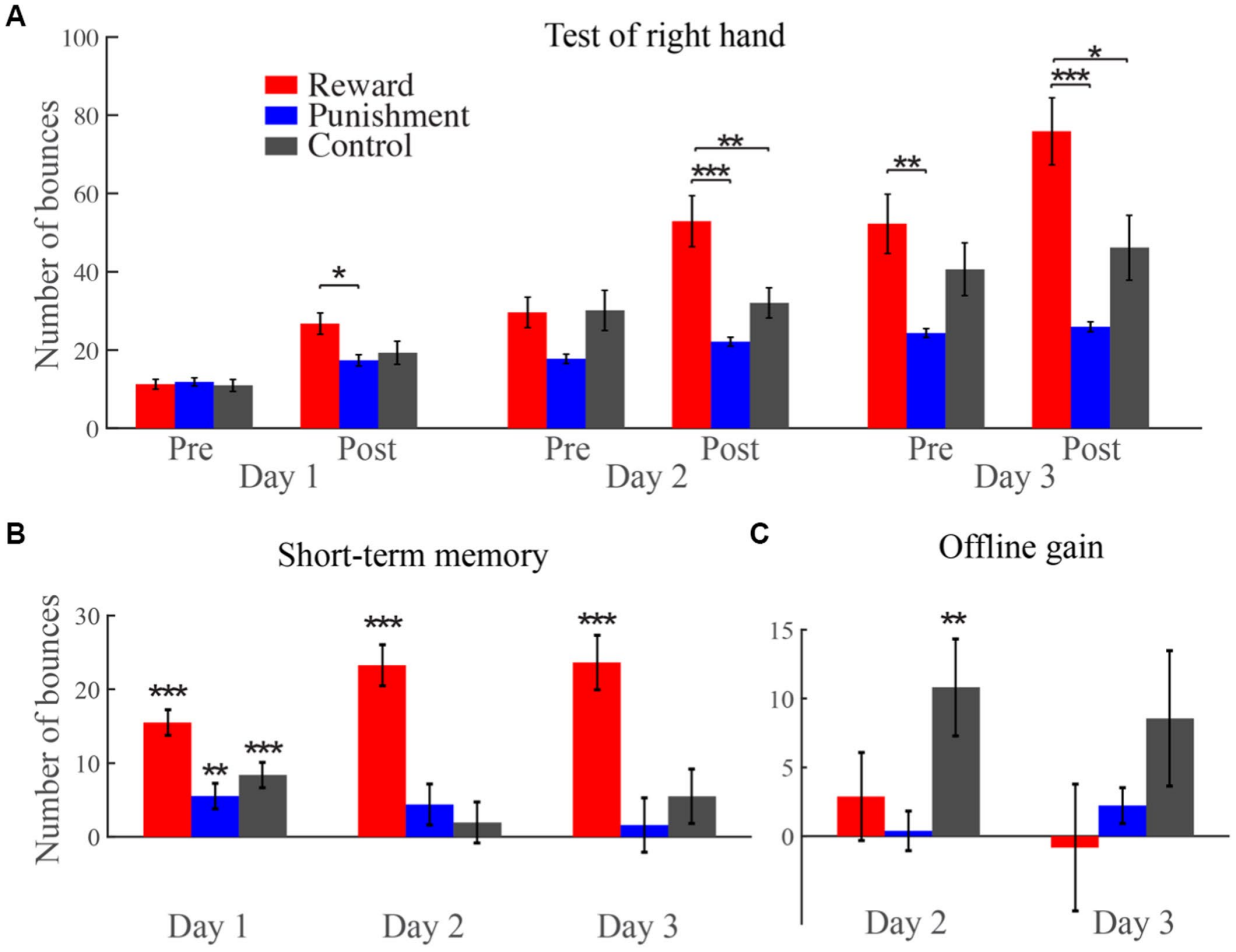
The number of ball bounces for the right hand during the pretest and posttest phases over the 3 days. **(A)** All data during the test phases for the right hand. **(B)** Comparison of performance between the two test phases for each day. **(C)** The offline memory gains on day 2 and day 3. *p < 0.05, **p < 0.01, ***p < 0.001. The same is below.

On day 1, after training with additional reward and punishment feedback or no feedback, the posttest of the right hand was 26.8 ± 2.7, 17.4 ± 1.5, and 19.3 ± 3.0 for the reward, punishment, and control group, respectively. On day 2, the number of ball bounces during the pretest phase for the three groups was 29.6 ± 3.9, 17.8 ± 1.2, and 30.1 ± 5.1. The number increased to 52.9 ± 6.5, 22.1 ± 1.14, and 32.1 ± 3.9 after training with reward, punishment or no feedback for the three groups, respectively. On day 3, pretest performance was 52.3 ± 7.6, 24.3 ± 1.1, and 40.6 ± 6.7, and posttest performance increased to 75.9 ± 8.6, 25.9 ± 1.3, and 46.1 ± 8.3, respectively (Figure 3A).

A 3 (groups) × 3 (days) × 2 (tests) mixed-design ANOVA revealed that all the three main effects were significant [the main effect of group: *F*_(2, 45)_ = 9.33; *p* < 0.001, $\eta_{p}^{2}$ = 0.29; the main effect of day: *F*_(1.42, 82.34)_ = 63.17; *p* < 0.001, $\eta_{p}^{2}$ = 0.58; the main effect of phase: *F*_(1, 90)_ = 79.13; *p* < 0.001, $\eta_{p}^{2}$ = 0.64]. There was also a significant interaction between day and group [*F*_(2.84, 82.34)_ = 8.08; *p* < 0.001, $\eta_{p}^{2}$ = 0.26] and a significant interaction between phase and group [*F*_(2, 90)_ = 23.49; *p* < 0.001, $\eta_{p}^{2}$ = 0.51]. Importantly, the interaction among the three factors also reached significance [*F*_(3.66, 82.34)_ = 2.71; *p* = 0.04, $\eta_{p}^{2}$ = 0.11]. We then performed a pairwise comparison across conditions for the three factors.

To determine the effect of reward and punishment on motor performance without reinforcement, we directly compared the performance of the three groups for different test phases on the 3 days. We confirmed that all groups started from a similar level before training. During the posttest on day 1, the number of ball bounces was significantly greater for the reward group than for the punishment group (mean difference = 5.5, *p* = 0.03). On day 2, we found no difference among the three groups during the pretest. However, after training, the performance of the reward group was better than that of the punishment group (mean difference = 30.8, *p* < 0.001) and the control group (mean difference = 20.8, *p* = 0.01) on the posttest. On day 3, the reward group continued to outperform the punishment group (mean difference = 27.9, *p* = 0.01) during the pretest. After training, the reward group remained superior to the punishment group (mean difference = 49.3, *p* < 0.001) and the control group (mean difference = 29.8, *p* = 0.01) during the posttest. In conclusion, the reward group showed better performance than the punishment group during the posttest on all the 3 days and during the pretest on day 3. In addition, the reward group showed better performance than the control group during the posttest on day 2 and day 3.

To test the reinforcement effect on short-term motor memory, we compared the difference between the two test phases among different days for different groups (Figure 3B). For the reward group, whether on day 1 (mean difference = 15.5, *p* < 0.001), day 2 (mean difference = 23.3, *p* < 0.001), or day 3 (mean difference = 23.6, *p* < 0.001), the number of ball bounces during the posttest was greater than that during the pretest. However, for the punishment group (mean difference = 5.5, *p* = 0.002) and the control group (mean difference = 8.4, *p* < 0.001), the participants showed better performance after training only on the first day. On the last 2 days, the performance of the two groups showed no difference between pretest and posttest. These results suggest that reward has a continuous facilitating effect on short-term motor memory.

To test the reinforcement effect on short-term motor memory, we compared the performance among the 3 days for pretest and posttest for different groups. For the reward group, whether for the pretest (*p*s < 0.001), or the posttest phase (*p*s < 0.001), the number of ball bounces increased significantly from day to day. The control group showed a similar tendency to the reward group: for the pretest, the participants showed a significant (from day 1 to day 2, *p* < 0.001) or marginally significant increase (from day 2 to day 3, *p* = 0.053) over the 3 days; for the posttest, the participants showed a significant increase from day to day. For the punishment group, however, there was no significant difference among the 3 days for either the pretest or the posttest. These results suggest that punishment may interfere with the formation or expression of long-term motor memory.

Finally, we examined the offline memory gains on day 2 and day 3 for the three groups by subtracting the number of ball bounces during the posttest on day 1 from that during the pretest on day 2, and by subtracting the number of bounces during the posttest on Day 2 from that during the pretest on day 3 (Figure 3C). The offline gains on day 2 was 2.9 ± 3.2, 0.4 ± 1.4, and 10.8 ± 3.5 for the reward, the punishment and the control group, respectively, with only the control group showing significance [one-sample *t*-test compared to 0°, *t*_(15)_ = 3.07, *p* = 0.008, *d* = 0.77]. The offline gains from day 2 to day 3 was −0.6 ± 4.6, 2.2 ± 1.3, and 8.6 ± 4.9, respectively, and none of them showed a significant difference from 0. This suggests that both reward and punishment interfere with long-term memory gains from day 1 to day 2.

### 3.3 Transfer effect of reward and punishment from the right to the left hand

**Figure 4:**
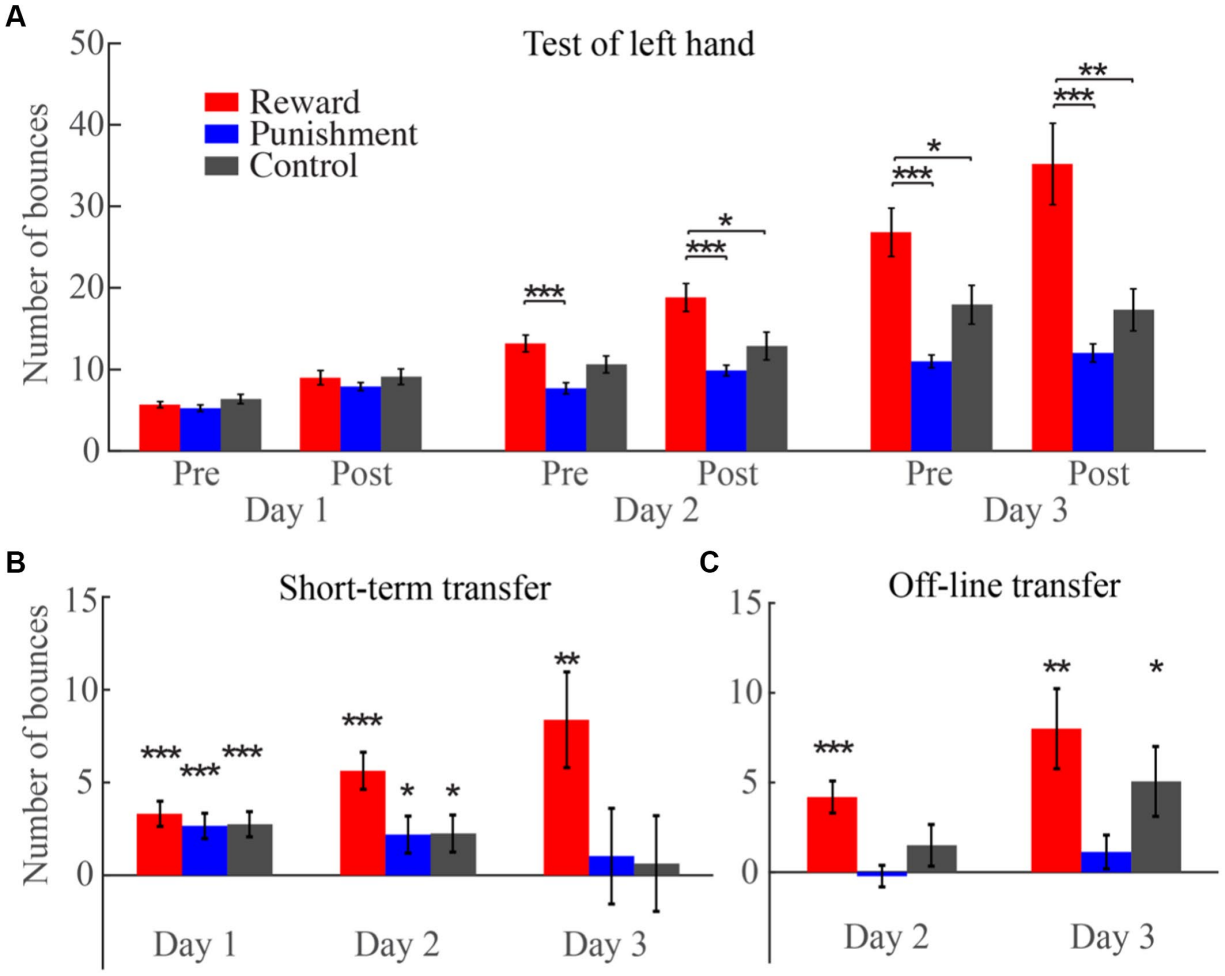
The number of ball bounces for the left hand during the pretest and posttest phases over the 3 days. **(A)** All data during the left-hand test phases. **(B)** The difference in left-hand performance between the two test phases for each day. **(C)** The offline gains in left-hand performance on day 2 and day 3.

In the following section, we sought to investigate whether the reinforcement given to the right-hand would affect the performance of the left hand. On day 1, the performance during the posttest was 9.0 ± 0.9, 7.9 ± 0.5, and 9.1 ± 1.0 for the reward, the punishment and the control group, respectively. On day 2, the number of ball bounces during the pretest was 13.2 ± 1.0, 7.7 ± 0.7, and 10.6 ± 1.0, while that during the posttest was 18.8 ± 1.7, 9.9 ± 0.7, and 12.9 ± 1.7, respectively. On day 3, the performance during the pretest was 26.8 ± 3.0, 11.0 ± 0.8, 17.9 ± 2.4, while that during the posttest was 35.2 ± 4.5, 12.0 ± 1.1, 17.3 ± 2.5 for the reward, the punishment and the control group, respectively (Figure 4A).

A 3 (groups) × 3 (days) × 2 (test phases) mixed-design ANOVA revealed that all the three main effects were significant [the main effect of group: *F*_(2, 45)_ = 14.02; *p* < 0.001, $\eta_{p}^{2}$ = 0.38; the main effect of day: *F*_(1.27, 57.33)_ = 76.95; *p* < 0.001, $\eta_{p}^{2}$ = 0.63; the main effect of phase: *F*_(1, 45)_ = 22.71; *p* < 0.001, $\eta_{p}^{2}$ = 0.34]. In addition, the interaction between day and group was significant: *F*_(2.55, 57.33)_ = 4.49; *p* < 0.001, $\eta_{p}^{2}$ = 0.40. The interaction between phase and group was significant: *F*_(2, 45)_ = 4.49; *p* = 0.017, $\eta_{p}^{2}$ = 0.17. The interaction between the three factors reached marginal significance: *F*_(2.57, 57.83)_ = 63.17; *p* = 0.094, $\eta_{p}^{2}$ = 0.09. We then performed a pairwise comparison across conditions for the three factors.

To determine the effect of reward and punishment on left hand performance, we directly compared the performance of the three groups between the two test phases for different days for the three groups. On day 1, no group difference was found for both test phases. On day 2, during the pretest phase, the reward group showed better performance than the punishment group (mean difference = 5.5, *p* < 0.001). During the posttest phase, the reward group performed better than the punishment group (mean difference = 8.9, *p* < 0.001) and the control group (mean difference = 5.9, *p* = 0.017). Importantly, the superiority of the reward group persisted in both test phases on day 3: the number of ball bounces for the reward group was greater than that for the punishment group (pretest: mean difference = 15.8, *p* < 0.001; posttest: mean difference = 23.2, *p* < 0.001) and the control group (pretest: mean difference = 8.9, *p* = 0.02; posttest: mean difference = 17.9, *p* = 0.001). This suggests that the facilitating effect of reward on the right hand has transferred to the left hand.

To test whether the reinforcement effect on short-term memory could transfer to the left hand, we compared between pretest and posttest among different days for each group (Figure 4B). For the reward group, the number of ball bounces during the posttest phase was greater than that during the pretest phase for all the 3 days (day 1, mean difference = 3.3, *p* < 0.001; day 2, mean difference = 5.6, *p* < 0.001; day 3, mean difference = 8.4, *p* = 0.002). For the punishment group, performance in the posttest phase was better than in the pretest phase only for the first 2 days (day 1, mean difference = 2.7, *p* < 0.001; day 2, mean difference = 2.2, *p* = 0.034), while performance did not improve after training on day 3. The control group showed a similar tendency to the punishment group: performance improved from pretest to posttest on day 1 (mean difference = 3.3, *p* < 0.001) and day 2 (mean difference = 5.6, *p* < 0.001), but not on day 3. This again suggests that the effect of reward on short-term memory transfers from the right to the left hand.

To test whether the reinforcement effect on long-term memory could transfer to the left hand, we compared the performance among different days for different groups during both test phases. For the reward group, the number of ball bounces increased day by day, regardless of the pretest and the posttest phases (*p*s < 0.001). The control group showed a similar tendency to the reward group. However, for the punishment group, the number of ball bounces did not show a significant difference among the 3 days for both test phases. This suggests that the negative effect of punishment on long-term memory transfers to the left hand.

Furthermore, we examined the offline left-hand gains on day 2 and day 3 for the three groups by subtracting the number of ball bounces during the posttest on day 1 from that during the pretest on day 2, and by subtracting the number of bounces during the posttest on day 2 from that during the pretest on day 3 (Figure 4C). The left-hand gains on day 2 was 4.2 ± 0.9, −0.2 ± 0.6, and 1.5 ± 1.2 for the reward, the punishment, and the control group, respectively, with only the reward group showing significance [one-sample *t*-test compared to 0°, *t*_(15)_ = 4.70, *p* < 0.001, *d* = 1.18]. The left-hand gains on day 3 was 8.0 ± 2.2, 1.1 ± 0.9, and 5.1 ± 2.0 for the three groups and only the reward group [*t*_(15)_ = 3.58, *p* = 0.003, *d* = 0.90] and the control group [*t*_(15)_ = 2.60, *p* = 0.02, *d* = 0.65] showed significant gains. This suggests that reward has an additional positive effect on left-hand gains on day 2 and further confirms that the negative effect of punishment on long-term memory gains transfers to the left hand.

## 4 Discussion

Our results confirmed our hypothesis by showing that reward and punishment have differential effects on the performance of real-world motor skill learning. Moreover, the effects change with training and readily transfer to novel contexts. Specifically, we found that reward led to better online learning during late training and had a persistent facilitating effect on short-term motor memory. Punishment led to better online learning during early training, but impaired long-term motor memory. Both reinforcements interfered with the long-term memory gains. Interestingly, the effects of reward and punishment on the right hand could transfer to the left hand.

Over the past few decades, researchers have compared the effects of performance-contingent monetary gains and losses on motor performance, which has been characterized by a simple heuristic: punishment benefits online performance, while reward benefits memory retention (Chen et al., 2018; Steel et al., 2020). These findings are typically based on simple laboratory motor tasks, such as, visuomotor adaptation and SRTT. However, neither of these can be considered as models of motor skill acquisition, defined as the incremental improvement in our ability to rapidly select and then precisely execute appropriate actions (Krakauer et al., 2019). Indeed, real-world motor skills are complex and may require thousands of hours of practice. Thus, it is largely unknown whether the effects of reward and punishment found in simple motor tasks extent to real-world motor skill learning.

As hand reaching and keyboard pressing are well-practiced actions in everyday life, there is no need for participants to build a new motor controller from scratch in visuomotor adaptation and SRTT. However, participants without a background in table tennis must learn to coordinate their muscles to bounce the ping-pong ball. During the ball bouncing task, participants learn to select the best point, time and force to hit the ball, improve their wrist rotation speed, and execute the movements both precisely and rapidly. In contrast to the two exemplar motor learning tasks, performance in the ping-pong ball bouncing task can hardly improve within a single training session. Therefore, participants in the present study were trained for three consecutive days. In the following sections, we will discuss our new findings using the novel task.

### 4.1 Punishment promoted early learning, while reward promoted late learning

We found that reward and punishment worked at different stages of learning: punishment promoted learning on day 1, while reward promoted learning on day 3. This result is partially consistent with previous results showing that punishment enhances online motor performance in motor adaption (Galea et al., 2015; Yin et al., 2023a,b) and SRTT (Wächter et al., 2009; Steel et al., 2016), where participants were only reinforced for 1 day. The facilitation effect of punishment can be explained by the asymmetric subjective value function in the prospect theory: the curve is generally steeper for losses than for gains (Kahneman and Tversky, 1979). That is, losing 10 points may have caused much stronger feelings than gaining 10 points, and thus people showed loss aversion. Particularly in early learning, when participants were not skilled, participants in the punishment group faced large losses, which may have a more pronounced motivational effect than reward. In sports settings, negative feedback is thought to induce a tendency to be more self-focused, causing participants to adopt a more attentional mode of control (Wulf and Lewthwaite, 2016). This control could motivate greater effort and energy expenditure (Nicholls et al., 2008; Gucciardi et al., 2009; Kaiseler et al., 2009; Ede et al., 2017). False-negative social comparative feedback has been found to improve performance in snooker (Welsh et al., 2023) and improve movement precision when learning an arm movement sequence (Zobe et al., 2019).

However, the promoting effect of punishment does not persist into day 2 and day 3, which could be explained by the argument that the effect of punishment tends to be short-lived (Skinner, 1965; Gershoff, 2002). In addition, punishment has non-negligible undesirable side effects. For example, when used excessively in sports training, punishment can promote the fear of failure, thereby increasing the likelihood of failure (Burton and Raedeke, 2008; Williams and Krane, 2014). In the present study, the continuous punishment during the three consecutive days may frustrate participants and decrease their self-efficacy during late learning (García et al., 2019).

On the contrary, although reward had no apparent facilitation effect during early learning, it facilitated late learning. The differential reinforcement effects during early and late training may be explained by the change in incentive size over the 3 days. Incentive size has been found to modulate the effect of reinforcement on motor learning (Adkins et al., 2021). Specifically, online performance was found to improve with increasing reward and punishment values. Compared to previous studies, only large reward can promote online performance for both motor adaptation (Nikooyan and Ahmed, 2015) and SRTT (Adkins et al., 2021). In the present study, the reward participants received larger reward, while the punishment participants received smaller punishment, with an improvement in performance from day 1 to day 3. It is possible that only large incentive size could promote online performance, regardless of reward or punishment. Future studies should examine the effect of incentive size on motor learning to confirm this hypothesis.

Almost any real-world motor task necessarily involves both cognitive and motor components. In most cases, explicit cognitive processes dominate early learning, while implicit motor execution processes dominate late learning (Krakauer et al., 2019). Therefore, we speculate that punishment benefits the formation of explicit cognitive processes, while reward benefits the implicit motor execution processes. This is supported by electroencephalography studies suggesting that punishment reflects an emphasized cognitive need for behavioral adjustments (Hamel et al., 2018), and that punishment, but not reward, modulates motor preparation (Hill et al., 2021), during which cognitive knowledge is largely involved.

Importantly, this speculation could be used to explain many controversial results found in previous studies. For example, as performance on the force tracking task (FTT) relies on more precise motor control and less explicit knowledge than motor adaptation, SRTT, and the task in the present study, punishment does not promote its online performance (Abe et al., 2011; Steel et al., 2016). When a perturbation is introduced gradually, participants typically adapt to it with little involvement of explicit knowledge (Yin and Wei, 2020). In this case, punishment does not benefit the online performance of the implicit adaptation process (Hamel et al., 2021). In contrast, when a motor adaptation task or SRTT is practiced for the first time, explicit processes may dominate the task. Therefore, many previous studies do not find a benefit of reward on online performance of motor adaptation and SRTT for single-session training (Abe et al., 2011; Galea et al., 2015; Steel et al., 2016; Yin et al., 2023a). It is possible that the benefit of reward emerges with longer-term training, when the implicit component predominates. This has been confirmed by relearning of a visuomotor adaptation (Song and Smiley-Oyen, 2017) and a reinforcement-based task, but in the new direction (Yin et al., 2023b).

The results suggest that reward and punishment may engage different neural mechanisms during real-world motor skill learning and provide preliminary evidence that the effect of reinforcement on motor learning may not be stable over time, but change dynamically with training. The results are consistent with our recent findings showing that punishment leads to faster initial learning, while reward promotes relearning in novel contexts in reinforcement-based motor learning (Yin et al., 2023b). In motor adaptation, we recently found that reinforcement with first punishment and then reward provided advantages over reinforcement with constant punishment or constant reward (Yin et al., 2023a). All these suggest that reward and punishment may benefit different learning processes and have advantages over different stages of motor learning. The dynamic change in the effect of reinforcement on online performance needs further investigation.

### 4.2 Reward facilitated short-term memory, while punishment impaired long-term memory

Interestingly, although reward did not improve online performance on the first 2 days, it improved posttest performance on all 3 days, which may be due to the spontaneous changes in brain activity following the reward (Steel et al., 2019). This is consistent with previous studies suggesting that reward promotes short-term memory retention in both motor adaptation (Galea et al., 2015; Quattrocchi et al., 2017) and SRTT (Wächter et al., 2009; Wilkinson et al., 2015). However, in contrast to the classic study (Abe et al., 2011), we did not find a benefit of reward on long-term memory across days in the present study, suggesting a different neural mechanism underlying the formation and consolidation of motor skill memory.

Although punishment facilitated learning on day 1, it impaired long-term memory across days. The present result contrasts with previous literature that finds no effect of punishment on long-term motor skill memory (Abe et al., 2011; Steel et al., 2016), but resonate well with studies in motor adaptation (Hamel et al., 2021). This phenomenon is consistent with the fact that conditions which foster rapid skill acquisition can impair long-term skill retention (Schmidt and Bjork, 1992). We speculate that the monetary loss provided a stressful learning context for the punishment group, which negatively impacted motor skill consolidation. It has been noted that even reward can impair spatial memory retention assessed 24 h after initial acquisition, as the participants were feared of being in a stressful learning context (Stamm et al., 2014).

### 4.3 Both reward and punishment interfered with long-term memory gains

Although not replicated by Steel et al. (2016), reward is found to lead to significant offline gains 24 h after the acquisition, and the gains could be maintained for at least 30 days (Abe et al., 2011). In the present study, we found significant memory gains from posttest on day 1 to pretest on day 2 only for the control group, suggesting that both reward and punishment interfere with long-term memory gains. The long-term memory gains in the control group could be explained by encoding specificity (Tulving and Thomson, 1973) or transfer-appropriate processing (Morris et al., 1977) since the context between the training and the test phases were the same, whereas the reinforcement groups would not benefit from this. In addition, the gain impairment could be explained by the undermining effect, which describes the phenomenon that training without reinforcement may have enhanced intrinsic motivation, whereas training with reinforcement discouraged it (Deci et al., 1999; Murayama et al., 2010; Cerasoli et al., 2014). The undermining effect may have caused participants in the reward and punishment groups to be less motivated than participants in the control group during the pretest on Day 2, when no reinforcement was imposed.

On the other hand, the frequency of feedback during training, one of the most important variables determining motor skill learning, could also explain the impairment of reinforcement on memory gains. There is considerable evidence that reducing the frequency of feedback leads to better motor memory retention (Wulf and Schmidt, 1989; Winstein and Schmidt, 1990). This is consistent with the animal studies of Pavlovian conditioning and instrumental learning showing that 100% reward leads to faster acquisition but 50% reward leads to slower extinction (Padilla, 1967; Prados et al., 2008). The retention advantage of partial reinforcement is also supported by visuomotor skill (Dayan et al., 2014) and adaptation tasks (Song and Smiley-Oyen, 2017; Hamel et al., 2019). In the present study, the provision of reward or punishment after each individual trial may induce a type of reinforcement reliance that leads to poor memory gain, especially when feedback is removed during the test phases.

### 4.4 The effects of reward and punishment transferred to the left hand

More intriguingly, we found that the effect of reward and punishment on the right hand could transfer to the left hand. Specifically, participants in the reward group showed better left-hand performance than the other two groups from the posttest on day 2. Reward facilitated left-hand posttest performance relative to pretest on all 3 days, whereas the other two groups facilitated left-hand posttest performance only on the first 2 days. In addition, punishment impaired long-term left-hand performance compared to the other two groups.

Few studies have examined the transfer effect of reward and punishment to other contexts. One exception comes from our lab which tests the transfer effect of reward and punishment in motor adaptation (Yin et al., 2023a). Specifically, we find that only the effect of reward and punishment combination can transfer to visuomotor rotation of the opposite direction. Neither the effect of pure reward, not pure punishment could transfer to motor adaptation. However, in the present study, we found that both the effects of reward and punishment could transfer from the right hand to the left hand in a real-world motor skill learning task. It has been reported that reward can increase both mental and physical effort (Schmidt et al., 2012) and it is plausible that the increased effort could not only improve the performance of the trained condition, but also improve performance in the untrained conditions. Motor skill learning is more effort-driven compared to motor adaption, where the intrinsic learning component plays a large role (Mazzoni and Krakauer, 2006), especially during late learning (Taylor et al., 2014). Therefore, we did not find a transfer effect of pure reward in motor adaptation (Yin et al., 2023a), but in motor skill learning.

In terms of punishment, the negative effect on long-term right-hand performance extended to the left hand. Although participants were never punished for their left-hand performance, the fear of failure induced by the stressful context inevitably impairs performance in both the trained and untrained conditions. Because the left-hand test follows closely on the heels of the right-hand test, the transfer became relatively easier than the condition in which the transfer is tested far from the trained condition (Yin et al., 2023a).

Importantly, the transfer of reinforcement effects across effectors suggests a regulatory mechanism for motor control that operates at a higher level than the motor learning of individual effectors. This could be explained by the metacognitive process of controlling and monitoring motor learning enabled by the reinforcement learning (Sugiyama et al., 2023). The researchers note that participants regulate their learning and retention rates to maximize reward and minimize punishment. In the present study, although reward and punishment are not used to directly regulate motor learning, the additional reinforcement feedback may influence the motor control policy that apple not only to the trained effector, but also to the untrained effector. Our findings suggest that metacognitive processes may be enabled not only by reinforcement-based motor learning, but also by additional reinforcement feedback during motor skill learning.

### 4.5 Limitations and future directions

Although the current study provides a novel understanding of the underlying learning process that occurs during a real-world motor skill task with reward and punishment, it has a number of limitations. First, we operationalize long-term memory as the difference in performance during the test phases between different days, which may confound the effects of learning and memory for the consecutive training design. In future studies, long-term memory should be tested after a longer time interval, such as 1 week or 1 month after the three-day training, to better understand the reinforcement effects on long-term memory retention.

Second, the exact reward and punishment contingencies can have a large impact on the results. It has been shown that reward is not effective in shaping motor behavior if participants are not aware of the manipulation being rewarded (Manley et al., 2014) or if the reward is too abundant (van der Kooij et al., 2018). The uniform point system cannot guarantee that all participants are optimally motivated. Furthermore, the effects depend on the characteristics of the participants (Quattrocchi et al., 2017; Huang et al., 2018). Future studies could use the inventory method to explore the psychological mechanism underlying the effect of reward and punishment.

Third, in additional to different learning processes, the schedule of reinforcement differs across tasks. For simple discrete motor skills, such as reaching adaptation (Galea et al., 2015), reinforcement can be delivered immediately after each movement. However, for continuous motor skills, such as SRTT (Wächter et al., 2009), FTT (Abe et al., 2011), locomotor adaptation (Hill et al., 2024), and the task we used in the present study, reinforcement could only be imposed after a series of movements. Whether and how the reinforcement schedule influences the reinforcement effects on different types of motor skills remains to be investigated.

Finally, as in most previous studies (Wächter et al., 2009; Abe et al., 2011; Galea et al., 2015), the punishment we used here is a type of negative punishment (removal of a positive stimulus), rather than positive punishment (addition of a negative stimulus, such as giving criticism), as defined by Skinner (1969). Future studies should test whether the two types of punishment have different effects on motor skill learning.

## 5 Conclusion

In conclusion, we show that reward and punishment have differential effects on real-world motor skill learning. The effect of reinforcement changes with training and transfers readily to new contexts. The results suggest that reward and punishment may act on different learning processes and engage different neural mechanisms during real-world motor skill learning. In addition, high-level metacognitive processes may be enabled by the additional reinforcement feedback during real-world motor skill learning. Our findings provide new insights into the mechanisms underlying motor learning, and may have important implications for practical applications such as sports training and motor rehabilitation.

## Data availability statement

The raw data supporting the conclusions of this article will be made available by the authors, without undue reservation.

## Ethics statement

The studies involving humans were approved by Capital University of Physical Education and Sports. The studies were conducted in accordance with the local legislation and institutional requirements. Written informed consent for participation in this study was provided by the participants. Written informed consent was obtained from the individual for the publication of any identifiable images included in this article.

## Author contributions

CY: Conceptualization, Data curation, Formal analysis, Funding acquisition, Investigation, Methodology, Project administration, Resources, Software, Supervision, Validation, Visualization, Writing – original draft, Writing – review & editing. YW: Conceptualization, Methodology, Resources, Writing – original draft, Writing – review & editing. BL: Conceptualization, Methodology, Resources, Writing – original draft, Writing – review & editing. TG: Conceptualization, Methodology, Resources, Writing – original draft, Writing – review & editing.
